# Predictive modeling of biomedical temporal data in healthcare applications: review and future directions

**DOI:** 10.3389/fphys.2024.1386760

**Published:** 2024-10-15

**Authors:** Abhidnya Patharkar, Fulin Cai, Firas Al-Hindawi, Teresa Wu

**Affiliations:** ^1^ School of Computing and Augmented Intelligence, Arizona State University, Tempe, AZ, United States; ^2^ ASU-Mayo Center for Innovative Imaging, Arizona State University, Tempe, AZ, United States

**Keywords:** biomedical temporal data, biomedical data challenges, forecasting, clinical predictive modeling, temporal data modeling problems, statistical time-series models, temporal machine learning models, deep temporal models

## Abstract

Predictive modeling of clinical time series data is challenging due to various factors. One such difficulty is the existence of missing values, which leads to irregular data. Another challenge is capturing correlations across multiple dimensions in order to achieve accurate predictions. Additionally, it is essential to take into account the temporal structure, which includes both short-term and long-term recurrent patterns, to gain a comprehensive understanding of disease progression and to make accurate predictions for personalized healthcare. In critical situations, models that can make multi-step ahead predictions are essential for early detection. This review emphasizes the need for forecasting models that can effectively address the aforementioned challenges. The selection of models must also take into account the data-related constraints during the modeling process. Time series models can be divided into statistical, machine learning, and deep learning models. This review concentrates on the main models within these categories, discussing their capability to tackle the mentioned challenges. Furthermore, this paper provides a brief overview of a technique aimed at mitigating the limitations of a specific model to enhance its suitability for clinical prediction. It also explores ensemble forecasting methods designed to merge the strengths of various models while reducing their respective weaknesses, and finally discusses hierarchical models. Apart from the technical details provided in this document, there are certain aspects in predictive modeling research that have arisen as possible obstacles in implementing models using biomedical data. These obstacles are discussed leading to the future prospects of model building with artificial intelligence in healthcare domain.

## 1 Introduction

### 1.1 Biomedical time series data

Clinical or biomedical data advances medical research by providing insights into patient health, disease progression, and treatment efficacy. It underpins new diagnostics, therapies, and personalized medicine, improving outcomes and understanding complex conditions. In predictive modeling, biomedical data is categorized as spatial, temporal, and spatio-temporal (Khalique et al., 2020; Veneri et al., 2012). Temporal data is key, capturing health evolution over time and offering insights into disease progression and treatment effectiveness. Time series data, collected at successive time points, shows complex patterns with short- and long-term dependencies, crucial for forecasting and analysis (Zou et al., 2019; Lai et al., 2018). Properly harnessed, this data advances personalized medicine and treatment optimization, making it essential in contemporary research.

### 1.2 Applications of predictive modeling in biomedical time series analysis

Predictive modeling with artificial intelligence (AI) has gained significant traction across various domains, including manufacturing (Altarazi et al., 2019), heat transfer (Al-Hindawi et al., 2023; 2024), energy systems (Huang et al., 2024), and notably, the biomedical field (Cai et al., 2023; Patharkar et al., 2024). Predictive modeling in biomedical time series data involves various approaches for specific predictions and data characteristics. Forecasting models predict future outcomes based on historical data, such as forecasting blood glucose levels for diabetic patients using past measurements, insulin doses, and dietary information (Plis et al., 2014). Classification models predict categorical outcomes, like detecting cardiac arrhythmias from ECG data by classifying segments into categories such as normal, atrial fibrillation, or other arrhythmias, aiding in early diagnosis and treatment (Daydulo et al., 2023; Zhou et al., 2019; Chuah and Fu, 2007). Anomaly detection in biomedical time series identifies outliers or abnormal patterns, signifying unusual events or conditions. For example, monitoring ICU patients’ vital signs can detect early signs of sepsis (Mollura et al., 2021; Shashikumar et al., 2017; Mitra and Ashraf, 2018), enabling timely intervention.

Table 1 summarizes the example applications of these models within the context of biomedical time series.

**TABLE 1:** Overview of predictive modeling techniques for biomedical time series and their example applications across healthcare scenarios.

Model type	Description	Bio-medical application example
Forecasting	Predicts a continuous value based on historical data	Predicting blood glucose levels for diabetic patients using past glucose measurements, insulin doses, and dietary information to forecast potential hypo- or hyperglycemic events (Plis et al., 2014)
Classification	Predicts categorical outcomes based on temporal data	Detecting cardiac arrhythmias (such as normal, atrial fibrillation, or other arrhythmias) (Daydulo et al., 2023; Zhou et al., 2019; Chuah and Fu, 2007)
Anomaly Detection	Identifies outliers or abnormal patterns within time series data	Sepsis detection. (Mollura et al., 2021; Shashikumar et al., 2017; Mitra and Ashraf, 2018)

### 1.3 Challenges in biomedical time series data

Regardless of the particular medical application or predictive model type used, models that manage biomedical time series data must tackle the intrinsic challenges posed by clinical and biomedical data. This includes various categories, such as electronic health records (EHRs), administrative data, claims data, patient/disease registries, health surveys, and clinical trials data. As illustrated in Table 2, each biomedical data category presents distinct challenges regarding quality, privacy, and completeness. During predictive modeling, further challenges arise. Specifically, we will investigate problems associated with missing data and imputation methods, the intricate nature of high-dimensional temporal relationships, and factors concerning the size of the dataset. Addressing these issues is crucial for developing strong and accurate predictive models in medical research.

**TABLE 2:** Overview of clinical data types and challenges. This table lists the main types of clinical and biomedical data, their definitions, and key challenges.

Data type	Definition	Challenges
Electronic Health Records (EHRs)	Digital records of patients medical history, treatments, and outcomes	• Data Standardization: Different formats across providers • Data Quality: Missing, incomplete, or inaccurate data • Privacy and Security: Ensuring compliance with regulations like HIPAA • Interoperability: Difficulties in data exchange between systems
Administrative Data	Data related to healthcare administration, such as hospital admissions and discharge records	• Limited Clinical Detail: Lack of in-depth clinical information • Data Timeliness: Potential delays in data availability • Standardization Issues: Variability in recording and categorization • Privacy Concerns: Maintaining patient confidentiality
Claims Data	Data from insurance claims used for billing and reimbursement	• Purpose and Detail: Primarily for billing, may lack clinical details • Lag Time: Delays between care and data availability • Coding Errors: Inaccuracies in coding (e.g., ICD codes) • Complexity: Requires specialized knowledge for interpretation
Patient/Disease Registries	Databases that track patients with specific conditions or diseases	• Data Completeness: Ensuring all relevant data is captured • Data Standardization: Different definitions and methods across registries • Funding and Maintenance: Need for consistent resources • Privacy Issues: Protecting patient confidentiality
Health Surveys	Data collected from health-related surveys and questionnaires	• Response Bias: Non-response or inaccurate self-reporting • Sampling Issues: Ensuring representative samples • Data Quality: Depends on survey design and execution • Timeliness: Time-consuming design, conduct, and analysis
Clinical Trials Data	Data from controlled trials testing the efficacy of treatments or interventions	• Complexity and Cost: Expensive and logistically complex • Regulatory Hurdles: Compliance with regulatory requirements • Data Sharing: Balancing patient confidentiality and proprietary interests • Generalizability: Trial participants may not represent the broader population

#### 1.3.1 Challenges in handling missing values and imputation methods in biomedical time series

Clinical data is often confronted with the issue of missing values, which can be caused by irregular data collection schedules or unexpected events (Xu et al., 2020). Medical measurements, recorded variably and at different times, may be absent, not documented, or affected by recording errors (Mulyadi et al., 2022), which makes the data irregular. Dealing with missing values in data sets usually involves either directly modeling data sets with missing values or filling in the missing values (a.k.a. imputation) to complete datasets for traditional analysis methods using data imputation techniques.

Current imputation techniques can be divided into four categories: case deletion, basic statistical imputation, machine learning-based imputation (Luo et al., 2018), and aggregating irregularly sampled data into discrete time periods (Ghaderi et al., 2023). Each of these methods comes with specific challenges in the context of handling biomedical temporal data. The deletion or omission of cases may lead to the loss of important information, particularly when the rate of missingness is high, which is critical in sensitive applications such as biomedical predictive modeling, where data is scarce and human lives are at risk. However, in certain cases, it is possible to do data omission without any potential risk to the outcome of the study. For instance, (Pinto et al., 2022), employs interrupted time series analysis to assess the impact of the “Syphilis No!” initiative in reducing congenital syphilis rates in Brazil. The results indicate significant declines in priority municipalities after the intervention. The study showcases the efficacy of public health interventions in modifying disease trends using statistical analysis of temporal data. Data collection needed to be conducted consistently over time and at evenly spaced intervals for proper analysis. To prevent bias due to the COVID-19 pandemic, December 2019 was set as the final data collection point, encompassing 20 months before the intervention (September 2016 to April 2018) and 20 months after the intervention (May 2018 to December 2019). This approach illustrates how the author addressed potential issues of irregular data or missing values in this context.

Contrary to data omission, statistical imputation techniques, such as mean or median imputation offer an alternative that reduces the effect of missing data, however, such methods do not take into account the temporal information but rather offer a summarized statistical imputation that often does not provide accurate replacement of the missing data. This could be critical in biomedical applications with scarce datasets, where the weight of a single data point could heavily affect the predictive power of the model. The use of machine learning-based imputation methods, such as Maximum Likelihood Expectation-Maximization, k-Nearest Neighbors, and Matrix Factorization, might offer a more accurate imputation that takes into account the specificity of the data point contrary to statistical aggregation methods, however, many of them still do not consider temporal relations between observations (Luo et al., 2018; Jun et al., 2019), and they usually are computationally expensive. Furthermore, without incorporating domain knowledge, these approaches can introduce bias and lead to invalid conclusions. Both machine learning and statistical techniques may not consider data distribution or variable relationships and may fail to capture complex patterns in multivariate time-series data due to the neglect of correlated variables, potentially resulting in underestimated or overestimated imputed values (Jun et al., 2019). Additionally, in real-time clinical decision support systems, timely and accurate data is crucial, as delays or errors in imputation can lead to incorrect decisions that directly affect patient outcomes. These systems demand high-speed processing, requiring imputation algorithms to be both computationally efficient and accurate. Moreover, the dynamic nature of clinical environments, where patient conditions can change rapidly, necessitates imputation methods that can adapt quickly to evolving data.

Aggregating measurements into discrete time periods can address irregular intervals, but it may lead to a loss of granular information (Ghaderi et al., 2023). Additionally, in time series prediction, missing values and their patterns are often correlated with target labels, referred to as informative missingness (Che et al., 2018). These limitations make it ill-advised to ignore, impute, or aggregate these values when handling biomedical time series data, but rather employ a model that is capable of handling the sparsity and the irregularity of clinical time series data.

#### 1.3.2 Complexities of high-dimensional temporal dependencies in biomedical data

Besides missing data challenges, hospitalized patients have a wide range of clinical events that are recorded in their electronic health records (EHRs). EHRs contain two different kinds of data: structured information, like diagnoses, treatments, medication prescriptions, vital signs, and laboratory tests, and unstructured data, like clinical notes and physiological signals (Xie et al., 2022; Lee and Hauskrecht, 2021), making them multivariate or high-dimensional (Niu et al., 2022).

The complexity of the relationships existing in such high-dimensional multivariate time series data can be difficult to capture and analyze. Analysts often try to predict future outcomes based on past data, and the accuracy of these predictions depends on how well the interdependencies between the various series are modeled (Shih et al., 2019). It is often beneficial to consider all relevant variables together rather than focusing on individual variables to build a prediction model, as this provides a comprehensive understanding of correlations in multivariate time series (MTS) data (Du et al., 2020). It thus becomes a requirement for predictive models employed in biomedical applications to take into account correlations among multiple dimensions and make predictions accordingly. It is equally crucial to ensure that only the features with a direct impact on the outcome are considered in the analysis. For instance, the study by Barreto et al. (2023) investigates the deployment of machine learning and deep learning models to forecast patient outcomes and allocate beds efficiently during the COVID-19 crisis in Rio Grande do Norte, Brazil. Out of 20 available features, nine were chosen based on their clinical importance and their correlation with patient outcomes, selected through discussions with clinical experts to guarantee the model’s accuracy and interpretability.

In addition to the inherent high dimensionality of biomedical data sourced from diverse platforms such as EHRs, wearable devices monitoring neurophysiological functions, and intensive care units tracking disease progression through physiological measurements (Allam et al., 2021), also display a natural temporal ordering. This temporal structure demands a specialized analytical approach distinct from that applied to non-temporal datasets (Zou et al., 2019). The temporal dependency adds significant complexity to modeling due to the presence of two distinct recurring patterns: short-term and long-term. For instance, short-term patterns may repeat daily, whereas long-term patterns might span quarterly or yearly intervals within the time series (Lai et al., 2018). Biomedical data often exhibit long-term dependencies, such as those seen in biosignals like electroencephalograms (EEGs) and electrocardiograms (ECGs), which may span tens of thousands of time steps or involve specific medical conditions such as acute kidney injury (AKI) leading to subsequent dialysis (Sun et al., 2021; Lee and Hauskrecht, 2021). Concurrently, short-term dependencies can manifest in immediate physiological responses to medical interventions, such as the administration of norepinephrine and subsequent changes in blood pressure (Lee and Hauskrecht, 2021). Another instance is presented by Valentim et al. (2022), who have created a model to forecast congenital syphilis (CS) cases in Brazil based on maternal syphilis (MS) incidences. The model takes into account the probability of proper diagnosis and treatment during prenatal care. It integrates short-term dependencies by assessing the immediate effects of prenatal care on birth outcomes, and long-term dependencies by analyzing syphilis case trends over a 10-year period. This strategy aids in enhancing public health decision-making and syphilis prevention planning.

Analyzing these recurrent patterns and longitudinal structures in biomedical data is essential to facilitate the creation of time-based patient trajectory representations of health events that facilitate more precise disease progression modeling and personalized treatment predictions (Allam et al., 2021; Xie et al., 2022). By incorporating both short-term fluctuations and long-term trends, robust predictive models can uncover hidden patterns in patient health records, advancing our understanding and application of digital medicine. Failing to consider these recurrent patterns can undermine the accuracy of time series forecasting in biomedical contexts such as digital medicine, which involves continuous recording of health events over time.

Additionally, early detection of diseases is of paramount importance. This can be achieved by utilizing existing biomarkers along with advanced predictive modeling techniques, or by introducing new biomarkers or devices aimed at early disease detection. For instance, early diagnosis of osteoporosis is essential to mitigate the significant socioeconomic impacts of fractures and hospitalizations. The novel device, Osseus, as cited by Albuquerque et al. (2023), addresses this by offering a cost-effective, portable screening method that uses electromagnetic waves. Osseus measures signal attenuation through the patient's middle finger to predict changes in bone mineral density with the assistance of machine learning models. The advantages of using Osseus include enhanced accessibility to osteoporosis screening, reduced healthcare costs, and improved patient quality of life through timely intervention.

#### 1.3.3 Dataset size considerations

The quantity of data available in a given dataset must be carefully considered, as it significantly influences model selection and overall analytical approach. For instance, when patients are admitted for brief periods, the clinical sequences generated are often fewer than 50 data points (Liu, 2016). Similarly, the number of data points for specific tests, such as mean corpuscular hemoglobin concentration (MCHC) lab results, can be limited due to the high cost of these tests, often resulting in less than 50 data points (Liu and Hauskrecht, 2015). Such limited data points pose challenges for predictive modeling, as models must be robust enough to derive meaningful insights from small samples without overfitting.

Conversely, some datasets may have a moderate sample length, ranging from 55 to 100 data points, such as the Physionet sepsis dataset (Reyna et al., 2019; 2020; Goldberger et al., 2000). These moderate-sized datasets offer a balanced scenario where the data is sufficient to train more complex models, but still requires careful handling to avoid overfitting and ensure generalizability.

In other cases, datasets can be extensive, particularly when long-span time series data is collected via sensor devices. These devices continuously monitor physiological parameters, resulting in large datasets with thousands of time steps (Liu, 2016). For example, wearable devices tracking neurophysiological functions or intensive care unit monitors can generate vast amounts of data, providing a rich source of information for predictive modeling. However, handling such large datasets demands models that are computationally efficient and capable of capturing long-term dependencies and complex patterns within the data.

The amount of data available is a major factor in choosing the appropriate model. Sparse datasets require models that can effectively handle limited information, often necessitating advanced techniques for data augmentation and imputation to make the most out of available data. Moderate datasets allow for the application of more sophisticated models, including machine learning and deep learning techniques, provided they are carefully tuned to prevent overfitting. Large datasets, on the other hand, enable the use of highly complex models, such as deep neural networks, which can leverage the extensive data to uncover intricate patterns and relationships.

### 1.4 Strategies in forecasting for biomedical time series data

While our discussion has generally revolved around the challenges in predictive modeling of biomedical temporal data, this review specifically emphasizes forecasting. From the earlier discourse, it is clear that a forecasting model for clinical or biomedical temporal data needs to adeptly manage missing, irregular, sparse, and multivariate data, while also considering its temporal properties and the capacity to model both short-term and long-term dependencies. The model should be able to make multi-step predictions, and the selection of a suitable model is determined by the amount of data available and the temporal length of the time series under consideration.

In this review, we initially examine three main categories of forecasting models: statistical, machine learning, and deep learning models. We look closely at the leading models within each category, assessing their ability to tackle the complexities of biomedical temporal data, including issues like data irregularity, sparsity, and the need to capture detailed temporal dependencies, alongside multi-step predictions. Since each category has its unique advantages as well as limitations in addressing the specific challenges of biomedical temporal datasets, other sets of models mentioned in the literature, known as hierarchical time series forecasting and combination or ensemble forecasting that merge the benefits of various forecasting models to produce more accurate forecasts are also covered.

The rest of the paper is structured as follows: In Section 2, statistical models are introduced. Section 3 covers machine learning models, while Section 4 focuses on deep learning models. This is followed by Section 5, which is a discussion section that summarizes the findings, discusses ensemble as well as hierarchical models, and explores future directions for the application of AI in clinical datasets. Finally, Section 6 concludes the paper.

## 2 Statistical models

The most popular predictive statistical models for temporal data are Auto-Regressive Integrated Moving Average (ARIMA) models, Exponential Weighted Moving Average (EWMA) models, and Regression models which are reviewed in the following sections.

### 2.1 Auto-Regressive Integrated Moving Average models

(Yule, 1927) proposed an autoregressive (AR) model, and (Wold, 1948) introduced the Moving Averaging (MA) model, which were later combined by Box and Jenkins into the ARMA model (Janacek, 2010) for modeling stationary time series. The ARIMA model, an extension of ARMA, incorporates differencing to make the time series stationary before forecasting, represented by ARIMA (p,d,q), where p is the number of autoregressive terms, d is the degree of differencing, and q is the number of moving average terms. ARIMA models have been applied in real-world scenarios, such as predicting COVID-19 cases. Ding et al. (2020) used an ARIMA (1,1,2) model to forecast COVID-19 in Italy. In another study, (Bayyurt and Bayyurt, 2020), utilized ARIMA models for predictions in Italy, Turkey, and Spain, achieving a Mean Absolute Percentage Error (MAPE) value below 10%. Similarly, (Tandon et al., 2022), employed an ARIMA (2,2,2) model to forecast COVID-19 cases in India, reporting a MAPE of 5%, along with corresponding mean absolute deviation (MAD) and multiple seasonal decomposition (MSD) values.

When applying ARIMA models to biomedical data, we select the appropriate model using criteria like Akaike Information Criterion (AIC) or Bayesian information criterion (BIC), estimate parameters using tools like R or Python's statsmodels, and validate the model through residual analysis. ARIMA models are effective for univariate time series with clear patterns, supported by extensive documentation and software, but they require stationarity and may be less effective for data with complex seasonality. Moreover, if a time series exhibits long-term memory, ARIMA models may produce unreliable forecasts (Al Zahrani et al., 2020), signifying that they are inadequate for capturing long-term dependencies. Additionally, ARIMA models necessitate a minimum of 50 data points in the time series to generate accurate forecasts (Montgomery et al., 2015). Therefore, ARIMA models should not be used for biomedical data that require the modeling of long-term relationships or have a small number of data points.

Several extensions such as Seasonal ARIMA (SARIMA) have been introduced for addressing seasonality. For instance, the research by Liu et al. (2023) examined 10 years of inpatient data on Acute Mountain Sickness (AMS), uncovering evident periodicity and seasonality, thereby establishing its suitability for SARIMA modeling. The SARIMA model exhibited high accuracy for short-term forecasts, assisting in comprehending AMS trends and optimizing the allocation of medical resources. An additional extension of ARIMA, proposed for long-term forecasts, is ARFIMA. In the study by Qi et al. (2020), the Seasonal Autoregressive Fractionally Integrated Moving Average (SARFIMA) model was utilized to forecast the incidence of hemorrhagic fever with renal syndrome (HFRS). The SARFIMA model showed a better fit and forecasting accuracy compared to the SARIMA model, indicating its superior capability for early warning and control of infectious diseases by capturing long-range dependencies. Additionally, it is apparent that ARIMA models cannot incorporate exogenous variables. Therefore, a variation incorporating exogenous variables, known as the ARIMAX model, has been proposed. The study by Mahmudimanesh et al. (2022) applied the ARIMAX model to forecast cardiac and respiratory mortality in Tehran by analyzing the effects of air pollution and environmental factors. The key variables encompass air pollutants (CO, NO2, SO2, PM10) and environmental data (temperature, humidity). The ARIMAX model is selected for its capacity to include exogenous variables and manage non-static time series data.

For multi-step ahead forecasting in temporal prediction models, two methods exist. The first, known as the plug-in or iterated multi-step (IMS) prediction that involves successively using the single step predictor, treating each prediction as if it were an observed value to obtain the expected future value. The second approach is to create a direct multi-step (DMS) prediction as a function of the observations, and to select the coefficients in this predictor by minimizing the sum of squares of the multi-step forecast errors. Haywood and Wilson (2009) developed a test to decide which of two approaches is more dependable based on a given lead-time. In addition to this test, there are other ways to decide which technique is most suitable for forecasting multiple steps ahead. One of these methods can be used to decide the best choice for multi-step ahead prediction either for ARIMA or other types of models depending on the amount of historical data and the lead-time.

### 2.2 Exponential weighted moving average models

The EWMA method, based on Roberts (2000), uses first-order exponential smoothing as a linear combination of the current observation and the previous smoothed observation. The smoothed observation $\tilde{y_{t}}$ at time t is given by the equation $\tilde{y_{t}}=\lambda y_{t}+\left(1-\lambda \right)\tilde{y_{t-1}}$ , where λ is the weight assigned to the latest observation. This recursive equation requires an initial value $\tilde{y_{0}}$ . Common choices for $\tilde{y_{0}}$ include setting it equal to the first observation $y_{1}$ or the average of available data, depending on the expected changes in the process. The smoothing parameter λ is typically chosen by minimizing metrics such as Mean Squared Error (MSE) or MAPE (Montgomery et al., 2015).

Several modifications of simple exponential smoothing exist to account for trends and seasonal variations, such as Holt's method (Holt, 2004) and Holt-Winter's method (Winters, 1960). These can be used in either additive or multiplicative forms. For modeling and forecasting biomedical temporal data, the choice of method depends on the data characteristics. Holt's method is more appropriate for data with trends. On the other hand, EWMA is suitable for stationary or relatively stable data, making it effective in scenarios without a clear trend, such as certain biomedical measurements. For instance, Rachmat and Suhartono (2020) performed a comparative analysis of the simple exponential smoothing model and Holt’s method for forecasting the number of goods required in a hospital’s inpatient service, assessing performance using error percentage and MAD. Their findings indicated that the EWMA model outperformed Holt’s method, as it produced lower forecast errors. This outcome is logical since the historical data of hospitalized patients lack any discernible trend.

EWMA models are also intended for univariate, regularly-spaced temporal data, as demonstrated in the example above (Rachmat and Suhartono, 2020), which uses a single variable (number of goods) over a period of time as input for model construction. This model is not suitable for biomedical data that involves multiple variables influencing the forecast unless its extention for multivariate data is employed. As highlighted by De Gooijer and Hyndman (2006), there has been surprisingly little progress in developing multivariate versions of exponential smoothing methods for forecasting. Poloni and Sbrana (2015) attributes this to the challenges in parameter estimation for high-dimensional systems. Conventional multivariate maximum likelihood methods are prone to numerical convergence issues and high complexity, which escalate with model dimensionality. They propose a novel strategy that simplifies the high-dimensional maximum likelihood problem into several manageable univariate problems, rendering the algorithm largely unaffected by dimensionality.

EWMA models cannot directly handle data that is not evenly spaced, and thus cannot be used to directly model biomedical data with a large number of missing values without imputation. These models are capable of multi-step ahead prediction either through DMS or IMS approach. To emphasize long-range dependencies, the parameter λ can be set to a low value, while a higher value will give more importance to recent past value (Rabyk and Schmid, 2016). The range of λ values typically used for reasonable forecasting is 0.1–0.4, depending on the amount of historical data available for modeling (Montgomery et al., 2015).

### 2.3 Regression models

Several regression models are available, and in this discussion, we focus on two specific types: multiple linear regression (MLR) (Galton, 1886; Pearson, 1922; Pearson, 2023) and multiple polynomial regression (MPR) (Legendre, 1806; Gauss, 1823). These models are particularly relevant for biomedical data analysis as they accommodate the use of two or more variables to forecast values. In MLR, there is one continuous dependent variable and two or more independent variables, which may be either continuous or categorical. This model operates under the assumption of a linear relationship between the variables. On the other hand, MPR shares the same structure as MLR but differs in that it assumes a polynomial or non-linear relationship between the independent and dependent variables. This review provides examination of these two regression models.

#### 2.3.1 Multiple linear regression models

The estimated value of output y at time t , denoted as $y_{t}$ with a MLR model for a certain set of predictors is given by the following Equation 1.

$$y_{t}=X_{t}\beta +\epsilon_{t}$$ (1)

where, $X_{t}=\left(1,x_{1t},x_{2t},\ldots ,x_{kt}\right)$ is a vector of k explanatory variables at time t , $\beta ={\left(\beta_{0},\beta_{1},\ldots ,\beta_{k}\right)}^{T}$ are regression coefficients, and $\epsilon_{t}$ is a random error term at time t , t=1,…,N (Fang and Lahdelma, 2016). It can be solved with least squares method (Pearson, 1901) to obtain the regression coefficients.

$R^{2}$ value can be calculated to check the accuracy of model fitting. The value of $R^{2}$ that is closer to 1 indicates better model performance. Metrics such as Root Mean Squared Error (RMSE), Mean Absolute Percentage Error (MAPE), and Theil’s inequality coefficient (TIC) are commonly utilized to assess the forecasting model’s performance. While RMSE is scale-sensitive, MAPE and TIC are scale-insensitive. Lower values for these three metrics signify a well-fitting forecasting model.

Zhang et al. (2021) developed an MLR model aimed at being computationally efficient and accurate for forecasting blood glucose levels in individuals with type 1 diabetes. These MLR models can predict specific future intervals (e.g., 30 or 60 min ahead). The dataset is divided into training, validation, and testing subsets; missing values are handled using interpolation and forward filling, and the data is normalized for uniformity. The MLR model showed strong performance, especially in 60-min forward predictions, and was noted for its computational efficiency in comparison to deep learning models. It excelled in short-term time series forecasts with significant data variability, making it optimal for real-time clinical applications.

#### 2.3.2 Multiple polynomial regression models

The estimated value of $y_{t}$ with say a second-order MPR model for a certain set of predictors is given by the following Equation 2.

$$\begin{array}{c} y_{t}=\beta_{0}+\beta_{1}x_{1t}+\cdots +\beta_{n}x_{nt}+\beta_{n+1}{x_{1t}}^{2}+\beta_{n+2}x_{1t}x_{2t}+\cdots \\ \;+\;\beta_{2n}x_{1t}x_{nt}+\beta_{2n+1}{x_{2t}}^{2}+\beta_{2n+2}x_{2t}x_{3t}+\cdots +\epsilon \end{array}$$ (2)

where, $\beta_{1t}$ , $\beta_{2t}$ are regression coefficients, $x_{1t},x_{2t},\ldots ,x_{nt}$ are predictor variables, and ϵ is a random error. The ordinary least squares method (Legendre, 1806; Gauss, 1823) is applicable for solving this, similar to how it is used with MLR models. Furthermore, the evaluation metrics utilized for MLR are also suitable for MPR models.

Wu et al. (2021) utilized US COVID-19 data from January 22 to July 20 (2020), categorizing it into nationwide and state-level data sets. Positive cases were identified as Temporal Features (TF), whereas negative cases, total tests, and daily positive case increases were identified as Characteristic Features (CF). Various other features were employed in different manners, such as the daily increment of hospitalized COVID-19 patients. An MPR model was created for forecasting single-day outcomes. The model consisted of pre-processing and forecasting phases. The pre-processing phase included quantifying temporal dependency through time-window lag adjustment, selecting CFs, and performing bias correction. The forecasting phase involved developing MPR models on pre-processed data sets, tuning parameters, and employing cross-validation techniques to forecast daily positive cases based on state classification.

The various applications of multiple regression models stated above, linear or polynomial, reveal their inability to directly capture temporal patterns. Although these models can accommodate multiple input variables, their design limits them to forecasting a singular outcome with one model. One of the extentions proposed to tackle this problem is multivariate MLR (MVMLR). Suganya et al. (2020) employs MVMLR to forecast four continuous COVID-19 target variables (confirmed cases and death counts after one and 2 weeks) using cumulative confirmed cases and death counts as independent variables. The methodology includes data preprocessing, feature selection, and model evaluation using metrics like Accuracy, $R^{2}$ score, Mean Absolute Error (MAE), Mean Squared Error (MSE), and Root Mean Squared Error (RMSE).

It is clear from the design of the regression models that they are unable to process missing input data. Unless all the predictor variables are present or substituted, the value of the output variable cannot be determined. Therefore, it becomes essential to apply imputation techniques prior to employing the regression models for forecasting.

The regression models do not usually require a large amount of data; it has been demonstrated to be effective with as few as 15 data points per case (Filipow et al., 2023). Multi-step ahead prediction can be accomplished with either IMS or DMS approaches when dealing with temporal data like previous cases. Nonetheless, as mentioned previously, since these methods do not inherently capture temporal dependencies, forecasts can be generated as long as the temporal order is maintained while training, and testing the model.

## 3 Machine learning models

Many machine learning models are employed to construct forecasting models for temporal data sets. The most popular models for temporal data sets include Support vector regression (SVR), k-nearest neighbors regression (KNNR), Regression trees (Random forest regression [RFR]), Markov process (MP) models, Gaussian process (GP) models. We will examine these techniques in the following sections.

### 3.1 Support vector regression

The origin of Support Vector Machines (SVMs) can be traced back to Vapnik (1999). Initially, SVMs were designed to address the issue of classification, but they have since been extended to the realm of regression or forecasting problems (Vapnik et al., 1996). The SVR approach has the benefit of transforming a nonlinear problem into a linear one. This is done by mapping the data set x into a higher-dimensional, linear feature space. This allows linear regression to be performed on the new feature space. Various kernels are employed to convert non-linear data into linear data. The most commonly used are linear kernel, polynomial kernel, and radial basis or Gaussian kernel.

Upon transforming a nonlinear dataset x into a higher-dimensional, linear feature space, the prediction function f(x) is expressed by Equation 3.

$$f\left(x\right)=w^{T}\phi \left(x\right)+b$$ (3)

The SVR algorithm solves a nonlinear regression problem by transforming the training data $x_{i}$ (where i ranges from one to N , with N being the size of the training data set) into a new feature space, denoted by ϕ(x) . This transformation allows establishing a linear relationship between input and output, using the weight matrix w and bias matrix b to further refine the model.

In SVR, selecting optimal hyperparameters (C,ϵ) is crucial for accurate forecasting. The parameter C controls the balance between minimizing training error and generalization. A higher C reduces training errors but may overfit, while a lower C results in a smoother decision function, possibly sacrificing training accuracy. The parameter ϵ sets a tolerance margin where errors are not penalized, forming an ϵ -tube around predictions. A larger ϵ simplifies the model but may underfit, whereas a smaller ϵ provides more detail, potentially leading to overfitting. Optimal values for C and ϵ may require additional methods (Liu et al., 2021).

SVR is often combined with other algorithms for parameter optimization. Evolutionary algorithms frequently determine SVR parameters. For example, Hamdi et al. (2018) used a combination of SVR and differential evolution (DE) to predict blood glucose levels with continuous glucose monitoring (CGM) data. The DE algorithm was used to determine the optimal parameters of the SVR model, which was then built based on these parameters. The model was tested using real CGM data from 12 patients, and RMSE was used to evaluate its performance for different prediction horizons. The RMSE values obtained were 9.44, 10.78, 11.82, and 12.95 mg/dL for prediction horizons (PH) of 15, 30, 45, and 60 min, respectively. It should be noted that when these evolutionary algorithms are employed for determining parameters, SVR encounters notable disadvantages, including a propensity to get stuck in local minima (premature convergence).

Moreover, SVR can occasionally lack robustness, resulting in inconsistent outcomes. To mitigate these challenges, hybrid algorithms and innovative approaches are applied. For instance, Empirical Mode Decomposition (EMD) is employed to extract non-linear or non-stationary elements from the initial dataset. EMD facilitates the decomposition of data, thereby improving the effectiveness of the kernel function Fan et al. (2017).

Essentially, SVR is an effective method for dealing with MTS data (Zhang et al., 2019). SVR, which operates on regression-based extrapolation, fits a curve to the training data and then uses this curve to predict future samples. It allows for continuous predictions rather than only at fixed intervals, making it applicable to irregularly spaced time series (Godfrey and Gashler, 2017). Nonetheless, due to its structure, SVR struggles to capture complex temporal dependencies (Weerakody et al., 2021).

It is suitable for smaller data sets as the computational complexity of the problem increases with the size of the sample Liu et al. (2021). It excels at forecasting datasets with high dimensionality Gavrishchaka and Banerjee (2006) due to the advanced mapping capabilities of kernel functions Fan et al. (2017). Additionally, multi-step ahead prediction in the context of SVR’s application to temporal data can be achieved either with the DMS or IMS approach (Bao et al., 2014).

### 3.2 K-nearest neighbors regression

In 1951, Evelyn Fix and Joseph Hodges developed the KNN algorithm for discriminant examination analysis (Fix and Hodges, 1989). This algorithm was then extended to be used for regression or forecasting. The KNN method assumes that the current time series segment will evolve in the future in a similar way to a past time series segment (not necessarily a recent one) that has already been observed (Kantz and Schreiber, 2004). The task is thus to identify past segments of the time series that are similar to the present one according to a certain norm. Given a time series $y_{N}\left(N\right)$ with N samples, the segment made of the last m samples is denoted as $y_{M}\left(N\right)$ , reflecting the current disturbance pattern. The KNN algorithm searches for k past time series intervals most comparable to $y_{M}\left(N\right)$ within the memory $y_{N}\left(N\right)$ using various distance metrics. For each nearest neighbor, a following time series of length h is generated, known as prediction contributions. Forecasting can then be done using unweighted or weighted approaches. In the unweighted approach, the prediction is the mean of the prediction contributions. In the weighted approach, the prediction is a weighted average based on the distance of each nearest neighbor from the current segment. Weights are assigned inversely proportional to the distances.

Gopakumar et al. (2016) employed the KNN algorithm to forecast the total number of discharges from an open ward in an Australian hospital, which lacked real-time clinical data. To estimate the next-day discharge, they used the median of similar discharges from the past. The quality of the forecast was evaluated using the mean forecast error (MFE), MAE, symmetric MAE (SMAPE), and RMSE. The results of these metrics were reported to be 1.09, 2.88, 34.92%, and 3.84, respectively, with an MAE error improvement of 16.3% over the naive forecast.

KNN regression is viable for multivariate temporal datasets, as illustrated by Al-Qahtani and Crone (2013). Nevertheless, its forecasting accuracy diminishes as the dimensionality of the data escalates. Consequently, it is critical to meticulously select pertinent features that impact the target variable to enhance model performance.

KNN proves effective for irregular temporal datasets (Godfrey and Gashler, 2017) due to its ability to identify previous matching patterns rather than solely depending on recent data. This distinctive characteristic renders KNN regression a favored choice for imputing missing data (Aljuaid and Sasi, 2016) prior to initiating any forecasting. Furthermore, it excels in capturing seasonal variations or local trends, such as aligning the administration of a medication that elevates blood pressure with a low blood pressure condition. Conversely, its efficacy in identifying global trends is limited, particularly in scenarios like septic shock, where multiple health parameters progressively deteriorate over time (Weerakody et al., 2021).

The KNN algorithm necessitates distance computations for k-nearest neighbors. Selecting an appropriate distance metric aligned with the dataset's attributes is essential, with Euclidean distance being prevalent, though other metrics may be more suitable for specific datasets. Ehsani and Drabløs (2020) examines the impact of various distance measures on cancer data classification, using both common and novel measures, including Sobolev and Fisher distances. The findings reveal that novel measures, especially Sobolev, perform comparably to established measures.

As the size of the training dataset increases, the computational demands of the algorithm also rise. To mitigate this issue, approximate nearest neighbor search algorithms can be employed (Jones et al., 2011). Furthermore, the algorithm requires a large amount of data to accurately detect similar patterns. Several methods have been suggested to accelerate the process; for example, (Garcia et al., 2010), presented two GPU-based implementations of the brute-force kNN search algorithm using CUDA and CUBLAS, achieving speed-ups of up to 64X and 189X over the ANN C++ library on synthetic data.

Similarly to other forecasting models, KNN is applicable for multistep ahead predictions using strategies such as IMS or DMS (Martínez et al., 2019). It is imperative to thoroughly analyze the clinical application and characteristics of the clinical data prior to employing KNN regression for forecasting, given its unique attributes. Optimizing the number of neighbors (k) and the segment length (m) through cross-validation is crucial. Employing appropriate evaluation metrics (e.g., MFE, MAE, SMAPE, RMSE) is necessary to assess the model’s performance.

### 3.3 Random forest regression

Random Forests (RFs), introduced by Breiman (2001), are a widely-used forecasting data mining technique. According to Bou-Hamad and Jamali (2020), they are tree-based ensemble methods used for predicting either categorical (classification) or numerical (regression) responses. In the context of regression, known as Random Forest Regression (RFR), RF models strive to derive a prediction function f(x) that reduces the expected value of a loss function L(Y,f(X)) , with the output Y typically evaluated using the squared error loss. RFR builds on base learners, where each learner is a tree trained on bootstrap samples of the data. The final prediction is the average of all tree predictions as shown by Equation 4.

$$f\left(x\right)=\frac{1}{K}\overset{K}{\underset{k=1}{\sum }}l_{k}\left(x\right)$$ (4)

where K is the number of trees, and $l_{k}\left(x\right)$ is the k -th tree. Trees are constructed using binary recursive partitioning based on criteria such as MSE.

Zhao et al. (2019) developed a RFR model to forecast the future estimated glomerular filtration rate (eGFR) values of patients to predict the progression of Chronic Kidney Disease (CKD). The data set used was from a regional health system and included 120,495 patients from 2009 to 2017. The data was divided into three tables: eGFR, demographic, and disease information. The model was optimized through grid-search and showed good fit and accuracy in forecasting eGFR for 2015–2017 using the historical data from the past years. The forecasting accuracy decreased over time, indicating the importance of previous eGFR records. The model was successful in predicting CKD stages, with an average $R^{2}$ of 0.95, 88% Macro Recall, and 96% Macro Precision over 3 years.

The study presented in Zhao et al. (2019) indicates that RFR is effective for forecasting multivariate data. Another research by Hosseinzadeh et al. (2023) found that RFR performs better with multivariate data than with univariate data, especially when the features hold substantial information about the target. Research by Tyralis and Papacharalampous (2017) indicated that RF incorporating many predictor variables without selecting key features exhibited inferior performance relative to other methods. Conversely, optimized RF utilizing a more refined set of variables showed consistent reliability, highlighting the importance of thoughtful variable selection.

Similar to SVR, RFR is able to process non-linear information, although it does not have a specific design for capturing temporal patterns (Helmini et al., 2019). RFR is capable of handling irregular or missing data. El Mrabet et al. (2022) compared RFR for fault detection with Deep Neural Networks (DNNs), and found that RFR was more resilient to missing data than DNNs, showing its superior ability to manage missing values. To apply RFR to temporal data, it must be suitably modeled. As an example, Hosseinzadeh et al. (2023) has demonstrated one of the techniques, which involves forecasting stream flow by modeling the RFR as a supervised learning task with 24 months of input data and corresponding 24 months of output sequence. The construction of sequences involves going through the entire data set, shifting 1 month at a time. The study showed that extending the look-back window beyond a certain time frame decreases accuracy, indicating RFR’s difficulty in capturing long-term dependencies when used in temporal modeling context. For a forecasting window of 24 months, the look-back window must be at least 24 months to avoid an increase in MAPE. This implies that although RFR can be used for temporal modeling, its effectiveness is more in capturing short-term dependencies rather than long-term ones. The experiments conducted by Tyralis and Papacharalampous (2017) also support this, showing that utilizing a small number of recent variables as predictors during the fitting process significantly improves the RFR’s forecasting accuracy.

RFR can be used to forecast multiple steps ahead, similar to other regression models used for temporal forecasting (Alhnaity et al., 2021). Regarding data management, RFR necessitates a considerable volume of data to adjust its hyperparameters. It can swiftly handle such extensive datasets, leading to a more accurate model (Moon et al., 2018).

### 3.4 Markov process models

Two types of Markov Process (MP) models exist: Linear Dynamic System (LDS) and Hidden Markov Model (HMM). Both of these models are based on the same concept: a hidden state variable that changes according to Markovian dynamics can be measured. The learning and inference algorithms for both models are similar in structure. The only difference is that the HMM uses a discrete state variable with any type of dynamics and measurements, while the LDS uses a continuous state variable with linear-Gaussian dynamics and measurements. These models are discussed in more detail in the following sections.

#### 3.4.1 Linear dynamic system

LDS, introduced by Kalman (1963), models the dynamics of sequences using hidden states and discrete time. It assumes evenly spaced time intervals within sequences, where the state transition and state-observation probabilities are given by $q_{i}$ and $o_{i}$ respectively. These probabilities are determined by the Equations 5, 6.

$$q_{i}=Aq_{i-1}+\epsilon_{i}$$ (5)

$$o_{i}=Bq_{i}+\zeta_{i}$$ (6)

The terms A and B represent the transition and emission matrices, respectively, whereas $\epsilon_{i}$ and $\zeta_{i}$ denote Gaussian noise components. Specifically, the stochastic element $\epsilon_{i}$ adheres to a zero-mean Gaussian distribution $\epsilon_{i}∼N\left(0,\;P\right)$ , characterized by a zero-mean vector and covariance matrix P . On the other hand, the stochastic component $\zeta_{i}$ follows a zero-mean Gaussian distribution $\zeta_{i}∼N\left(0,\;R\right)$ , which is also characterized by a zero-mean vector and covariance matrix R . The initial state distribution $\left(q_{1}\right)$ is defined, with mean ξ and covariance matrix ψ , i.e., $q_{1}∼N\left(\xi ,\;\psi \right)$ . The set of LDS parameters is denoted as λ=(A,B,P,R,ξ,ψ) . In applied scenarios, these parameters necessitate estimation from empirical data. Two standard approaches for learning LDS are the Expectation-Maximization (EM) (Ghahramani and Hinton, 1996) and spectral learning algorithms (Katayama, 2005; Overchee and Moor, 1996; Doretto et al., 2003). EM iteratively maximizes the likelihood of observations by cycling between expectation (E-step) and maximization (M-step). It is precise but can be slow and prone to local optima, especially with limited training data. Spectral learning algorithms provide a non-iterative, closed-form solution using singular value decomposition (SVD) to estimate LDS parameters. They are faster but may be less precise than EM.

A new data-driven state-space dynamic model was developed by Wang et al. (2014) using an extended Kalman filter to estimate time-varying coefficients based on three variate time series data corresponding to glucose, insulin, and meal intake from type 1 diabetic subjects. This model was used to forecast blood glucose levels and was evaluated against a standard model (forgetting-factor-based recursive ARX). The results showed that the proposed model was superior in terms of fit, temporal gain, and J index, making it better for early detection of glucose trends. Furthermore, the model parameters could be estimated in real time, making it suitable for adaptive control. This model was tested for various prediction horizons, demonstrating the model’s suitability for multi-step ahead prediction.

The LDS is apt for modelling multivariate temporal data, yet it is confined to data sampled at regular time intervals. As a result, its application to irregularly spaced data (Shamout et al., 2021) or time series with missing values may be problematic. In such instances, modifications and extension are needed. For example, Liu et al. (2013) presented a novel probabilistic method for modeling clinical time series data that accommodates irregularly sampled observations using LDS combined with GP models. They defined the model by a series of GPs, each confined to a finite window, with dependencies between consecutive GPs represented via an LDS. Their experiments on real-world clinical time series data demonstrate that their model excels in modeling clinical time series and either outperforms or matches alternative time series prediction models.

Typically, implementing the LDS model starts with thorough data preparation, requiring uniform sampling. In cases of irregular sampling or datasets with missing values, proper management through interpolation or imputation is essential for using the model without alterations, as mentioned above. The model architecture is constructed using hidden state variables $\left(q_{i}\right)$ to encapsulate the latent processes, alongside measurable observation variables $\left(o_{i}\right)$ representing directly observable quantities. Parameters such as the state transition matrix (A), the emission matrix (B), and the covariance matrices for process noise (P) and observation noise (R) should be initialized based on prior knowledge or through randomization techniques. Parameter learning is facilitated through the EM algorithm or spectral learning methods, with practical considerations dictating the choice: EM being preferred for its precision with limited datasets and spectral methods for their computational expediency.

The LDS or Kalman filter remains a cornerstone for tracking and estimation due to its attributes of simplicity, optimality, tractability, and robustness. However, nonlinear system applications present complex challenges, often mitigated by the Extended Kalman Filter (EKF) (Lewis, 1986) which linearizes nonlinear models to leverage the linear Kalman filter. Also, various advancements have been proposed for LDS, particularly when addressing nonlinear or non-Gaussian dynamics. For example, approximate filtering methodologies such as the unscented Kalman filter (Julier and Uhlmann, 1997), alongside Monte Carlo-based techniques including the particle filter (Gordon et al., 1993) and the ensemble Kalman filter (Evensen, 1994), are also utilized similar to EKF. Model evaluation is conducted through cross-validation employing metrics such as MSE or RMSE. For forecasting applications, the model can be employed for one-step ahead forecasts or extended to iterative multi-step predictions.

#### 3.4.2 Hidden markov model

Hidden Markov Models (HMMs), introduced by Baum and colleagues in the late 1960s and early 1970s (Baum and Petrie, 1966; Baum and Eagon, 1967; Baum and Sell, 1968; Baum et al., 1970; Baum, 1972), are powerful tools for linking hidden states with observed events, assuming an underlying stochastic process. An HMM consists of a set of hidden states, a transition probability matrix, a sequence of observations, observation likelihoods, and an initial state distribution. A critical assumption in HMMs is output independence, where the probability of an observation depends solely on the state that produced it.

HMMs address three fundamental problems: (1) Likelihood estimation: Using the forward or backward algorithm to compute the probability of an observed sequence given the model parameters; (2) Decoding: Employing the Viterbi algorithm to determine the optimal sequence of hidden states corresponding to a sequence of observations; and (3) Learning: Applying the Baum-Welch algorithm, a special case of the EM algorithm, to estimate HMM parameters from observation sequences.

Sotoodeh and Ho (2019) proposed a novel feature representation based on the HMM to predict the length of stay of patients admitted to the ICU. This representation was composed of a specified time resolution and a summary statistic calculated for a specific time window for each feature (e.g., average, most recent, maximum, etc.). An HMM was then trained on these features, and used to generate a series of states for each patient, with the first and last states being used as it was thought that these could better explain the variance in the length of stay. This feature matrix was then used as the input to a regression model to estimate the length of stay. Experiments were conducted to determine the optimal number of states, overlapping or non-overlapping time windows, aggregation of ICU types, summary measure for each time window, and selection of time window probabilities. The model was compared to other baseline models, and was found to have a lower RMSE than all of them.

It is evident from the application here that HMM is capable of dealing with multivariate data. Additionally, it is designed to process temporal data that is spaced at regular intervals of time (Shamout et al., 2021). Unfortunately, it is not able to process temporal data that is irregular or has missing values. Cao et al. (2015) employed both DMS and IMS strategies to forecast multiple future system states and anticipate the evolution of a fault in the Tennessee Eastman (TE) chemical process using HMM. They reported the accuracy of 1,2,3,.,20 step-ahead predictions, which were similar for both approaches, with the DMS approach being slightly more accurate than the IMS approach. This is understandable, as the IMS approach has to contend with additional complexities, such as cumulative errors, decreased precision, and increased uncertainty. This demonstrates the capability of HMM to make predictions for multiple steps in the future.

HMM can be constructed using either raw time series data or extracted features. Samaee and Kobravi (2020) introduced a forecasting model aimed at forecasting the timing of tremor bursts with a nonlinear hidden Markov model. This model was trained using the Baum-Welch algorithm, employing both raw Electromyogram (EMG) data and extracted features such as integrated EMG, mean frequency, and peak frequency. The study found that an HMM trained on raw EMG data performed better at forecasting tremor occurrences, suggesting that raw data more accurately captures tremor dynamics compared to extracted features. This is likely due to the short time window being insufficient for feature-based methods. Therefore, it is crucial to determine whether raw time series data or extracted features yield better performance in HMM construction.

In general, MP models are well-recognized for their efficacy in capturing short-term relationships (Manaris et al., 2011) between adjacent symbols or sequences with strong inter-symbol ties. However, they prove inadequate for representing long-distance dependencies between symbols that are spatially or temporally distant (Yoon and Vaidyanathan, 2006; Manaris et al., 2011). To enhance the representational scope of these models, certain methodologies must be employed. For instance, Yoon and Vaidyanathan (2006) proposed context-sensitive HMMs capable of capturing long-distance dependencies, thereby enabling robust pairwise correlations between distant symbols.

Additionally, a limitation of Markov models is that the intrinsic dimensionality of its hidden states is not known beforehand. If the dimensionality is too large, there is a risk of the model becoming overfitted. Therefore, it is often necessary to try out different training sizes and intrinsic dimensionality of the hidden states to create a model that fits (Liu, 2016).

### 3.5 Gaussian process models

The Gaussian process (GP), introduced by Williams and Rasmussen (Williams and Rasmussen, 2006), is a non-parametric, non-linear Bayesian model in statistical machine learning. A GP is a collection of random variables, any finite number of which have a joint Gaussian distribution. This model extends the multivariate Gaussian to infinite-sized collections of real-valued variables, defining the distribution over random functions. A GP is represented by the mean function: m(x)=E[f(x)] , and the covariance function: $K^{G}\left(x,x\prime \right)=E\left[\left(f\left(x\right)-m\left(x\right)\right)\left(f\left(x\prime \right)-m\left(x\prime \right)\right)\right]$ , where f(x) is a real-valued process and, x and x′ are two input vectors.

In the context of biomedical temporal data, GP shows promises for modeling and forecasting due to their flexibility and ability to incorporate uncertainty. For example, GP can be used to model patient vital signs over time or predict disease progression (Siami-Namini et al., 2019). The key advantage of GP is their ability to provide uncertainty estimates along with predictions, which is crucial in biomedical applications where uncertainty quantification can inform clinical decisions. The GP can compute the distribution of function values for any set of inputs. This initial distribution, known as the prior, is a multivariate Gaussian represented by Equation 7.

$$f\left(X^{*}\right)∼N\left(m\left(X^{*}\right),\;K^{G}\left(X^{*},X^{*}\right)\right)$$ (7)

When given observed data, the GP updates this to the posterior distribution, which also follows a multivariate Gaussian. This updated distribution incorporates the observed data, providing more accurate predictions. The posterior distribution is influenced by the observed values and accounts for noise in the data.

GPs extend the multivariate Gaussian distribution into an infinite function space, making them suitable for time series modeling. They can handle observations taken at any time, whether regularly or irregularly spaced, and can make future predictions by calculating the posterior mean for any given time index. Additionally, GPs can act as non-linear transformation operators by replacing the linear transformations used in traditional temporal models with GP, offering a flexible approach to modeling complex data.

GP parameters consist of mean and covariance function parameters. The mean function, dependent on time, represents the expectation before observations. In cases of uncertain trend directions, constant-offset mean functions are common. If prior knowledge about the long-term trend exists, it can be incorporated into GP models, optimizing mean function parameters using gradient-based methods. In clinical scenarios with diverse patient ages and circumstances, aligning time origins is challenging. A practical approach is setting mean functions to a constant (m(t)=M) , making the GP time-invariant. The constant M is determined by averaging all patient observations. To optimize the covariance function parameters Θ , one can maximize the marginal likelihood p(Y|X) . The log marginal likelihood for GP is calculated where Y includes all training observations. The covariance matrix for noisy observations is represented by $K^{Y}$ . It is calculated as $K^{Y}=K^{G}+\sigma^{2}I$ , where $K^{G}$ is the covariance matrix for noise-free function values, and σ is a standard deviation of the noise, represented as, ϵ∼N(0,σ) . The partial derivatives of the marginal likelihood with respect to each parameter in Θ are then derived. These derivatives are used in gradient-based optimization methods to maximize p(Y|X) , thereby optimizing the covariance function parameters.

A prevalent limitation of GP models pertains to their high computational demands. Sparse GP methodologies have been devised to mitigate this challenge (Williams and Rasmussen, 2006; Quinonero-Candela and Rasmussen, 2005), primarily by identifying a subset of pseudo inputs to alleviate computational load. Further optimization of computational efficiency can be achieved through the application of the Kronecker product (Stegle et al., 2011), synchronization of training data across identical time intervals for each dimension (Evgeniou et al., 2005), or the implementation of recursive algorithms tailored for online settings (Pillonetto et al., 2008). Applications necessitating near real-time retraining are more apt to benefit from these approaches, whereas methods that extend over more prolonged temporal frameworks exhibit reduced sensitivity to such computational constraints. Another shortcoming of GP is that it models each time series separately, disregarding the interactions between multiple variables. To tackle this problem and capture the multivariate behavior of MTS, the multi-task Gaussian process (MTGP) was proposed (Bonilla et al., 2007).

#### 3.5.1 Multi-task Gaussian process

MTGP is an extension of GP that models multiple tasks (e.g., MTS) simultaneously by utilizing the learned covariance between related tasks. It uses $K^{C}$ to model the similarities between tasks and $K^{G}$ to capture the temporal dependence with respect to time stamps. The covariance function of MTGP is given by Equation 8.

$$K^{M}=K^{C}⊗K^{G}+D⊗I_{T}$$ (8)

where $K^{C}$ is a positive semi-definite matrix and $K_{j,k}^{C}$ measures the similarity between time series j and time series k. D is an n x n diagonal matrix in which $D_{j,j}$ is the noise variance ${\delta_{j}}^{2}$ for the $j^{th}$ time series. ⊗ is the Kronecker product.

The parameters of GP-based models are composed of parameters that define the mean and covariance functions. Generally, the covariance function ensures that values of the function for two close times tend to have a high covariance, while values from inputs that are distant in time usually have a low covariance. These parameters can be acquired from data that includes one or multiple examples of time series. The predictions of values at future times are equivalent to the calculation of the posterior distribution for those times.

Proper data preprocessing is essential when building MTGP models for forecasting time series. This involves transformations such as detrending and applying logarithmic adjustments. Methods like spectral mixture kernels or Bayesian Nonparametric Spectral Estimation can be employed for initialization. Post-training, it is vital to visualize and interpret cross-channel correlations to better understand the inherent patterns, thereby supporting practical and accurate forecasting applications (de Wolff et al., 2021).

Shukla (2017) proposed to use MTGP to forecast blood pressure from Photoplethysmogram (PPG) signals and compared its performance to Artificial Neural Networks (ANNs). Ten features were extracted from the PPG signal, and five of them were chosen as the tasks (or targets) to construct the MTGP model. These features were systolic blood pressure, diastolic blood pressure, systolic upstroke time, diastolic time and cardiac period. Four different ANN models were built based on one or more of the above tasks. The models were evaluated on clinical data from the MIMIC Database, with the absolute error e calculated for each heart beat as the performance measure. The results showed that the performance of MTGP was either comparable to or better than the ANNs and existing methods of computing BP from non-invasive data. MTGP is thus applicable for modeling multivariate temporal data with multiple prediction targets. In a study by Dürichen et al. (2014), MTGP was employed on three diverse biomedical data sets. The experiments aimed to illustrate that forecasting all correlated variables simultaneously enhanced prediction performance, contrasting with individual variable predictions. MTGP has been demonstrated to be successful in multi-step ahead forecasting for a variety of biomedical domain applications mentioned here, as well as in other domains (Cai et al., 2020).

GP models, with an appropriate choice of covariance function, can capture rapid changes in a time series and can be applied to time series modeling problems by representing observations as a function of time. This means that there is no restriction on when the observations are made or if they are regularly or irregularly spaced in time. Liu (2016) and Cheng et al. (2020) demonstrated that, with the appropriate selection of a covariance function, it is possible to model both the short-term dependencies or long-term correlations of temporal data. GP models also work well with small amounts of data (Liu, 2016). It is possible to predict with a certain degree of certainty (confidence interval) using GP (Roberts et al., 2013), which is usually essential for temporal modeling of medical data that necessitates a certain degree of assurance to be employed by medical professionals to make their decisions. However, this approach has some limitations, the most serious being that the mean function of the GP is a function of time and must be set to a constant value in order to make the GP independent of the time origin. This significantly restricts its ability to represent changes or different modes in time series dynamics.

## 4 Deep learning models

The use of Deep Learning techniques for predicting time series data has gained significant attention. While there are various models available for handling time-series data, in this review, we will focus on some commonly used models for forecasting clinical data sets over time. Specifically, we will explore Recurrent Neural Networks (RNN), Long Short Term Memory Networks (LSTM), and Transformer models.

### 4.1 Recurrent Neural Networks

**FIGURE 1:**
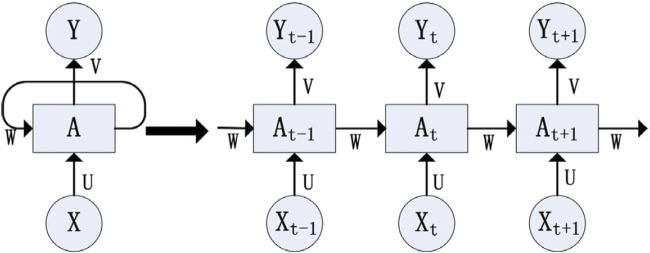
RNN structure (reproduced from Liu et al., 2021, licensed under CC BY 4.0).

The concept of RNN was introduced by Elman (1990) for identifying patterns in sequential data. RNNs accept sequential data as input and process it recursively. In an RNN, nodes are linked sequentially, where the input at time t depends on the output at time t−1 . The structure and functions of RNNs are depicted in Figure 1.

In this structure, the input layer (X) is weighted by U , the hidden layer (A) by W , and the output layer (Y) by V . The equations employed for calculations are Equations 9, 10.

$$Y_{t+1}=g\left(VA_{t+1}\right)$$ (9)

$$A_{t+1}=f\left(UX_{t+1}+WA_{t}\right)$$ (10)

The above formula is iterative in nature and can be expanded using the Equation 11 as:

$$Y_{t+1}=Vf\left(UX_{t+1}+Wf\left(UX_{t}+Wf\left(UX_{t-1}+\cdots \;\right)\right)\right)$$ (11)

The equation above demonstrates that the RNN network’s output $Y_{t+1}$ is influenced by the current input $A_{t+1}$ , as well as the previous inputs $A_{t},A_{t-1},.$ . RNNs effectively handle sequential and correlated data by considering historical inputs. The work of Chandra et al. (2021) demonstrates its applicability in multi-step ahead prediction. Although their demonstration focuses on univariate cases, RNN has also been successfully applied to multivariate cases. In their study, Zhu et al. (2020) utilized four data fields for each instance: sampling time, CGM values, meal intake, and insulin dose. They employed a deep learning approach using an extention of RNN, dilated RNN (DRNN), to forecast glucose levels for the next 30 min. The DRNN model exhibits superior performance compared to current models like autoregressive (ARX), SVR, and neural networks for glucose prediction (NNPG), when evaluated on the OhioT1DM dataset. The RMSE values reported are ARX: 20.1 mg/dL, SVR: 21.7 mg/dL, NNPG: 22.9 mg/dL, and DRNN: 18.9 mg/dL. RNNs are frequently used to handle missing values or irregularities in multivariate temporal datasets. There are two main approaches to achieve this: imputation and data generation, or a forecasting approach. When using the first approach, RNNs leverage temporal correlations within each series and correlations among multiple features to fill in missing values or create a time series that captures the original characteristics. On the other hand, the latter approach involves the development of more advanced RNN-based solutions that provide a deeper understanding of the missing data, as well as the patterns and relationships within the data (Weerakody et al., 2021).

Implementing RNNs for modeling and forecasting biomedical temporal data necessitates meticulous attention to data preprocessing, model structure, tuning of hyperparameters, and evaluation techniques. The recommendations for each aspect are outlined as discussed in Hewamalage et al. (2021). Deseasonalization is advised for datasets exhibiting seasonal trends unless consistent seasonal patterns exist, which RNNs can inherently manage. Data normalization enhances training convergence, while the sliding window approach divides the time series into overlapping sequences for model input. Hyperparameter tuning is crucial for achieving optimal RNN performance. Principal hyperparameters include the learning rate, batch size, and the number of layers. The learning rate must be selected judiciously; for ideal convergence, the Adagrad optimizer typically needs a higher learning rate ranging between 0.01 and 0.9, whereas the Adam optimizer performs effectively within a narrower range of 0.001–0.1. The batch size should be commensurate with the dataset size, and usually, one or two layers are sufficient, as additional layers may result in overfitting. Setting high values for the standard deviation of regularization parameters for Gaussian noise and L2 weight regularization can cause significant underfitting, reducing the neural network's efficacy in generating forecasts. One category of RNN models, stacked RNNs, which involve multiple RNN layers, are employed for forecasting and often utilize skip connections to alleviate vanishing gradient issues. Another category of RNN models, known as sequence-to-sequence (S2S) models, is typically applied in sequential data transformations and is useful for tasks like multi-step forecasting. Assessing RNN performance against traditional methods like ARIMA using standard metrics and cross-validation confirms their competitiveness. Enhancements to RNN methods, such as attention mechanisms and ensemble methods, further boost their performance. Attention mechanisms enable the model to concentrate on relevant parts of the input sequence, while ensemble methods combine several RNN models to produce robust forecasts, reducing biases and variances.

RNNs excel at capturing short-term dependencies (Helmini et al., 2019). They are more sensitive to time series data than traditional convolutional neural networks (CNNs) and can retain memory during data transmission. However, as previously mentioned, when the input sequence lengthens, the network demands more temporal references, leading to a deeper network. In longer sequences, it becomes challenging for the gradient to propagate back from later sequences to earlier ones, resulting in the vanishing gradient problem. Consequently, RNNs struggle with long-term dependencies. To mitigate this vanishing (or exploding) gradient issue, a modification of the RNN known as the long sshort-term memory (LSTM) model was introduced by Hochreiter and Schmidhuber (1997).

#### 4.1.1 Long Short Term Memory Networks

**FIGURE 2:**
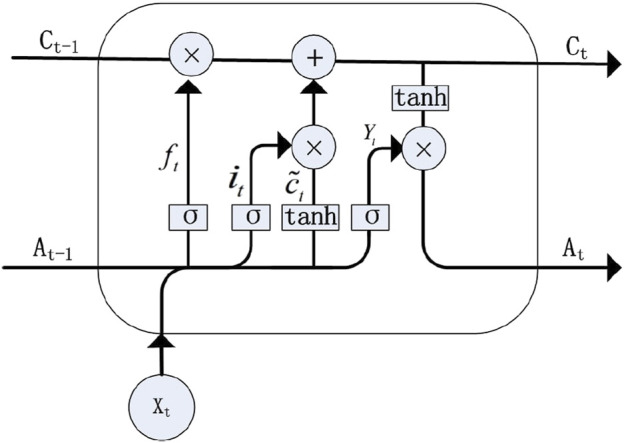
LSTM structure (reproduced from Liu et al., 2021, licensed under CC BY 4.0).

To overcome the challenges of vanishing and exploding gradients in RNNs, the LSTM model was introduced. This architecture employs a cell state to maintain long-term dependencies, as discussed by Helmini et al. (2019). The model effectively manages gradient dispersion by establishing a retention mechanism between input and feedback. Figure 2 illustrates the LSTM structure (Weerakody et al., 2021). Additionally, LSTM models are proficient in capturing short-term dependencies, primarily through the use of a hidden state. LSTM units are controlled by three gates: the input gate, the output gate, and the forget gate. These gates regulate the flow of information and maintain the cell state, enabling LSTMs to retain important information over long periods. The key equations (Equations 12–17) governing LSTM operations are mentioned as follows:

$$f_{t}=\sigma \left(W_{fA}A_{t-1}+W_{fX}X_{t}+b_{f}\right)$$ (12)

$$i_{t}=\sigma \left(W_{iA}A_{t-1}+W_{iX}X_{t}+b_{i}\right)$$ (13)

$$\tilde{c_{t}}=\tanh\left(W_{cA}A_{t-1}+W_{cX}X_{t}+b_{c}\right)$$ (14)

$$c_{t}=\left(f_{t\;}◦\;c_{t-1}\right)+\left(i_{t}\;◦\;{\tilde{c}}_{t}\right)$$ (15)

$$Y_{t}=\sigma \left(W_{YA}A_{t-1}+W_{YX}X_{t}+b_{Y}\right)$$ (16)

$$A_{t}=\left(Y_{t}\;◦\tanh\left(c_{t}\right)\right)$$ (17)

In these equations, σ represents the sigmoid function, and ◦ denotes element-wise multiplication. The forget gate $\left(f_{t}\right)$ controls the retention of the previous cell state $\left(c_{t-1}\right)$ , the input gate $\left(i_{t}\right)$ manages the incorporation of new information, and the output gate $\left(Y_{t}\right)$ determines the output based on the cell state $\left(c_{t}\right)$ . $W_{fA}$ , $W_{fX}$ , $W_{iA}$ , $W_{iX}$ , and $W_{cA}$ are different weights associated with the forget gate, input gate, and the current input unit state.

A deep learning neural network (NN) model based on LSTM with the addition of two fully connected layers was proposed by Idriss et al. (2019), for forecasting blood glucose levels. To determine the optimal parameters for the model, several experiments were conducted using data from 10 diabetic patients. The performance of the proposed LSTM NN, as measured by RMSE, was compared to that of a simple LSTM model and an autoregressive (AR) model. The results indicated that the LSTM NN achieved higher accuracy (mean RMSE = 12.38 mg/dL) compared to both the existing LSTM model (mean RMSE = 28.84 mg/dL) for all patients and the AR model (mean RMSE = 50.69 mg/dL) for 9 out of 10 patients. LSTM is therefore valuable in the representation of time-based information.

One popular extention of the LSTM network is a Bidirectional LSTM (BiLSTM) model which is obtained by modifying the architecture of the LSTM network to include two LSTM layers: one processing the input sequence from left to right (forward direction) and the other from right to left (backward direction). This bidirectional traversal allows the model to have information from both past and future contexts, enhancing its ability to capture complex patterns and dependencies. The outputs from both layers are concatenated at each time step, providing a richer representation of the input sequence. This approach results in improved performance for tasks like time series forecasting, as BiLSTM models can leverage additional training from both directions to better understand sequential data (Abbasimehr and Paki, 2022). For instance, in a study by Said et al. (2021), a bidirectional LSTM (Bi-LSTM) was employed to analyze multivariate data from countries grouped based on demographic, socioeconomic, and health sector indicators alongwith the information on lockdown measures, to predict the cumulative number of COVID-19 cases in Qatar from December 1st to 31 December 2020.

LSTM is also combined with multi-head attention mechanisms. This approach aims to address the non-linear patterns and complexities often found in real-world time series data, which traditional forecasting techniques struggle to predict accurately (Siami-Namini et al., 2019). When dealing with irregular temporal data that contain missing values, traditional LSTM models face challenges and may produce suboptimal analyses and predictions. This is because applying the LSTM model to irregular temporal data, either by filling in missing values or using temporal smoothing, does not enable the model to differentiate between actual observations and imputed values. Therefore, caution is advised when using an LSTM model on a dataset where multiple missing values have been imputed.

### 4.2 Transformer models

**FIGURE 3:**
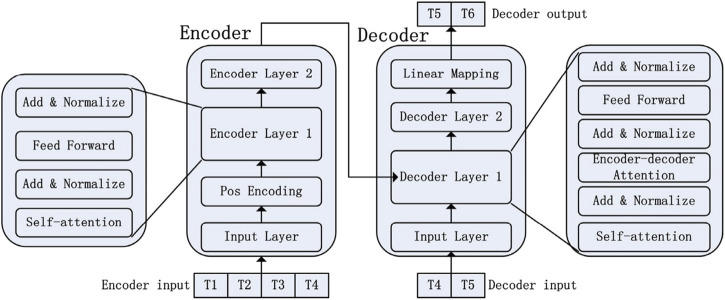
Transformer architecture (reproduced from Liu et al., 2021, licensed under CC BY 4.0).

The Transformer model for natural language processing (NLP) was introduced by Vaswani et al. (2017). This model is composed of an encoder-decoder network, which differs from the traditional sequential structure of RNN. Transformer model utilizes the Self-Attention mechanism to enable parallel training and capture global information. The encoder takes historical time series data as input, while the decoder predicts future values using an auto-regressive approach. This means that the decoder’s generated output at each step is based on previously generated outputs. To establish a connection between the encoder and decoder, an attention mechanism is employed. This allows the decoder to learn how to effectively focus (“pay attention”) on relevant parts of the historical time series before making predictions. The decoder utilizes masked self-attention to prevent the network from accessing future values during training, thereby avoiding information leakage. The typical architecture of the Transformer model is depicted in the Figure 3.

Originally designed for NLP tasks, the Transformer architecture has found application in temporal forecasting as well. To model irregular temporal data, various methods have been proposed. For instance, Tipirneni and Reddy (2022) introduced the Self-supervised Transformer for Time-Series (STraTS) model, which treats each time-series as observation triplets (time, variable, value) instead of matrices as done by conventional methods. This approach eliminates the need for aggregation or imputation. STraTS utilizes a Continuous Value Embedding (CVE) scheme to retain detailed time information without discretization.

The study by Harerimana et al. (2022) utilized a Multi-Headed Transformer (MHT) model to forecast clinical time-series variables from charted vital signs, leveraging the transformer architecture’s attention mechanism to capture complex temporal dependencies. The dataset is split into training and testing sets per patient, using past 24-h data for recursive future predictions. Training involves a fixed dimension of 512 for all layers, and the model is evaluated using metrics like Area under the Receiver Operating Characteristic Curve (AUC-ROC), MSE, and MAPE. The MHT model outperforms traditional models (LSTM, Temporal Convolutional Network, TimeNet) in forecasting vital signs, length of stay, and in-hospital mortality, demonstrating superior accuracy and robustness by focusing on influential past time steps, validating its efficacy in handling clinical time-series data.

The Transformer architecture is a relatively new concept, and ongoing research is being conducted to explore its capabilities. For instance, Li et al. (2019) suggest that unlike RNN-based methods, the Transformer enables the model to access any part of the time series history, disregarding the distance. This characteristic potentially makes it more adept at capturing recurring patterns with long-term dependencies. However, Zeng et al. (2023) presented an opposing viewpoint, questioning the effectiveness of Transformer-based solutions in long-term time series forecasting (LTSF). They argue that while Transformers are adept at capturing semantic correlations in sequences, their self-attention mechanism, which is invariant to permutations, may result in the loss of crucial temporal information necessary for accurate time series modeling. In support of their claim, the researchers introduced LTSF-Linear, a simple one-layer linear model, and discovered that it outperformed more complex Transformer-based LTSF models on nine real-life data sets. In addition, a temporal fusion transformer (TFT) was suggested by Zhang et al. (2022) as a method that effectively captures both short-term and long-term dependencies. Hence, when employing Transformer-based approaches for temporal forecasting, it is crucial to take into account these distinct viewpoints and conduct experiments to determine the most effective modeling technique for the specific forecasting task, considering the presence of short-term and long-term dependencies.

While DL models are capable of generating precise predictions, they are frequently perceived as black-box models that lack interpretability and transparency in their internal processes (Vellido, 2019). This presents a significant issue as medical professionals are often hesitant to trust machine recommendations without a clear understanding of the underlying rationale. In addition, significant quantities of clinical data are utilized to generate standardized inputs for training DL models. The challenge of acquiring extensive clinical data sets poses a challenge in the integration of DL clinical models into real-world clinical systems (Xiao et al., 2018).

## 5 Discussion

This section is comprised of two subsections. The first subsection summarizes the overview of the models and their capacities in addressing the difficulties encountered in forecasting of clinical datasets. The second subsection explores the future prospects concerning the practical obstacles in implementing AI models for biomedical data modeling.

### 5.1 Summary of models for biomedical temporal data forecasting

#### 5.1.1 Summary of statistical, ML, and DL models

This review focuses on predictive models for biomedical temporal data, which face several challenges such as missing values due to irregular data collection or errors. Traditional methods use imputation or deletion, but models that handle missing values without these steps are preferable, as patterns of missing data might hold valuable information termed as “informative missingness”. EHRs often feature MTS data, so models must capture these correlations. Temporal data complexity requires models to consider short-term and long-term patterns. Short-term patterns might involve events like norepinephrine administration linked to recent hypotension, while long-term patterns could involve past acute kidney injury necessitating dialysis. Models should account for these dependencies and support multi-step ahead forecasting for early disease detection. Data availability varies with clinical events, thus impacting model selection. These challenges are crucial for accurate, effective predictions in clinical settings. Table 3 summarizes the advantages and disadvantages of the discussed models, supplemented by literature insights.

**TABLE 3:** Advantages and disadvantages of models for handling biomedical temporal data.

Model type	Advantages	Disadvantages
ARIMA	- Captures linear dependencies and trends - Interpretable parameters - Works well with stationary data	- Needs data to be stationarized - Lacks ability to handle missing values - Inability to manage multivariate data - Can not capture long-range dependencies
EWMA	- Proficient in temporal modeling - Simple and computationally efficient - Adapts quickly to recent changes in data - Useful for smoothing noisy data - Effective in short-range modeling	- Not suitable for complex patterns - Unsuitable for handling multivariate data - Critical initialization and parameter selection - Capable of long-range modeling with parameter adjustment
MLR	- Interpretable coefficients - Insights into variables’ relationships - Performs well with small-mid datasets - Efficiently manages multivariate data - Can be adapted for temporal modeling	- Assumes linear relationships - Sensitive to multicollinearity - Requires features to be linearly related to the target.
MPR	- Can capture higher-order relationships - More flexible than MLR. - Suitable for polynomial relationships - Manages multivariate data efficiently	- Prone to overfitting with high polynomial-degrees - Interpretation of coefficients can be complex - Unable to handle missing values
SVR	- Effective in high-dimensional spaces - Can capture nonlinear relationships - Robust to overfitting with regularization - Manages multivariate data efficiently - Although not designed for temporal modeling, but can be adapted to capture them	- Computationally complex - Needs support from other algorithms for hyperparameter tuning - Lacks robustness resulting in inconsistent outcomes - Struggles to capture complex temporal dependencies - Memory intensive for large datasets
KNNR	- Non-parametric and flexible - Can be adapted for temporal modeling - Proficient in handling missing values - Efficiently manages multivariate data - Effective in short-range modeling due to its unique structure	- Expensive for large datasets - Memory intensive - Falls short in capturing global dependencies
RFR	- Handles nonlinear relationships - Robust to overfitting - Can handle high-dimensional data - Manages multivariate data efficiently - Capable of handling irregular or missing data	- Time consuming for large datasets - Requires careful tuning of hyperparameters - Difficulty in handling long-range dependencies
LDS	- Captures temporal dependencies - Efficiently handles multivariate data - Captures short-term relationships	- Complex parameter tuning - Cannot deal with irregular data - Difficulty with nonlinear relationships
HMM	- Captures hidden influencing states - Useful for sequential data modeling - Efficiently handles multivariate data - Can capture short-term dependencies efficiently	- Training complexity - Lacks interpretability of hidden states - Prone to overfitting when intrinsic dimensionality exceeds data - Struggles with capturing long-term dependencies
MTGP	- Models multiple tasks simultaneously - Captures correlations between tasks - Provides uncertainty estimates - Can forecast efficiently with irregular data - Flexible covariance function that can capture both short-range and long-range dependencies	- Complex to implement and tune - If the GP is made time independent, it restricts the representation of changes in time series dynamics - Computationally intensive on large-scale
RNN	- Proficient in handling missing values - Can handle variable-length sequences - Effective for multivariate sequential data modeling	- Vanishing/exploding gradient problem - Training can be slow - Difficulty with very long-term dependencies
LSTM	- Handles vanishing gradient problem - Captures long-term dependencies effectively - Robust to sequence length variations	- Training complexity - Lacks interpretability - Requires careful hyperparameters tuning - May produce suboptimal analyses and predictions when modeling imputed data
Transformer	- Highly suitable for multivariate temporal modeling - Parallel processing of sequences - Scalable to large datasets - Effective in short-range modeling	- Computationally intensive - Requires large amounts of data - Lacks interpretability - Fine-tuning can be complex - Uncertain effectiveness in managing long-term dependencies

Forecasting is categorized into statistical, ML, and DL methods. We focused on models frequently used in biomedical temporal modeling, evaluating their effectiveness. For statistical methods, we analyzed ARIMA, EWMA, and regression models. In ML, we assessed SVR, RFR, KNNR, MP, and GP models. For DL methods, we evaluated RNN, LSTM, and Transformer models. Our analysis found the MTGP model effective for irregularly spaced data, capturing both short-term and long-term dependencies with an appropriate covariance function. It predicts multiple steps ahead and accounts for autocorrelation within and correlation between time series, making it suitable for multivariate temporal analysis with small to moderate data. However, MTGP’s computational cost can be high with large data, and a constant mean function may limit its ability to represent time series dynamics. While MTGP is suitable for biomedical temporal modeling, alternative approaches include improving current models, adopting ensemble methods, or using hierarchical approaches discussed later in this paper.

Improving existing models by incorporating new techniques can address limitations in temporal analysis of biomedical data. For instance, while RNNs struggle with long-range dependencies, they handle other temporal challenges well. To overcome this, Zhu et al. (2020) introduced a dilated RNN, enhancing neuron receptive fields to capture long-term dependencies, enabling 30-min glucose level forecasts. Similarly, HMMs lack long-range correlation modeling. Yoon and Vaidyanathan (2006) introduced context-sensitive HMM (csHMM), capturing long-range correlations by adding context-sensitivity to model states. Additionally, the interpretability in DL models is essential. Tipirneni and Reddy (2022) proposed an interpretable model with outputs as linear combinations of individual feature components. Slight modifications to the original models can address specific limitations.

Even though various modifications have been suggested to address the shortcomings of individual models, certain limitations remain insurmountable. A recently emerging solution involves combining multiple models to create a fusion model, which allows for the integration of their strengths and mitigation of their weaknesses. These fusion models, also known as combination or ensemble forecasting models, is examined in the next subsection.

#### 5.1.2 Fusion models

A different approach to enhance forecasting precision involves merging multiple models, also known as combination or ensemble forecasting models. The paper by Wang et al. (Wang et al., 2023) provides a comprehensive overview of the evolution and effectiveness of combining multiple forecasts to enhance prediction accuracy. Combining forecasts, known as “ensemble forecasts,” integrates information from various sources, avoiding the need to identify a single “best” forecast amidst model uncertainty and complex data patterns. The review covers simple combination methods, such as equally weighted averages, which surprisingly often outperform more sophisticated techniques due to their robustness and lower risk of overfitting. Linear combinations, which determine optimal weights based on historical performance, and nonlinear combinations, which account for nonlinear relationships using methods like neural networks, are also discussed. Wang et al. (2023) emphasize the potential of learning-based combination methods, such as stacking and cross-learning, which improve accuracy by training meta-models on multiple time series. In stacking, several forecasting models are trained on the original dataset, and their predictions are combined by a meta-model to provide an optimal forecast. Cross-learning builds on this by utilizing data from various time series to train the meta-model. The review also highlights the crucial role of diversity and precision in forecast combinations, pointing out that successful combinations are enhanced by diverse individual forecasts.

These techniques have been successfully applied to biomedical data forecasting. For example, Naemi et al. (2020) introduced a customizable real-time hybrid model, leveraging the Nonlinear Autoregressive Exogenous (NARX) model along with Ensemble Learning (EL) (RFR and AdaBoost), to forecast patient severity during their stay at Emergency Departments (ED). This model makes use of patient vital signs such as Pulse Rate (PR), Respiratory Rate (RR), Arterial Blood Oxygen Saturation (SpO2), and Systolic Blood Pressure (SBP), which are recorded during treatment. The model forecasts the severity of illness in hospitalized patients at ED for the upcoming hour based on their vital signs from the previous 2 hours. The effectiveness of the NARX-EL models is evaluated against other baseline models including ARIMA, a fusion of NARX and LR, SVR, and KNNR. The findings revealed that the proposed hybrid models could predict patient severity with significantly higher accuracy. Furthermore, it was noted that the NARX-RF model excels at predicting abrupt changes and unexpected adverse events in patients’ vital signs, exhibiting an $R^{2}$ score of 0.978 and NRMSE of 6.16%. Kandula et al. (2018) used a super-ensemble technique to combine information from different forecasting methods robustly. This method yielded a more accurate comprehensive forecast on average than a single model. They compared three forecasting approaches for predicting seven characteristics of seasonal influenza during the 2016–2017 USA season: a mechanistic method, a weighted average of two statistical methods, and a super-ensemble of eight statistical and mechanistic models. The study found the meta-ensemble approach to be the most accurate overall. Katari et al. (2023) employed a combination of Decision Tree (DT) and Ada Boosting algorithms for heart disease prediction. The study highlights the importance of early diagnosis due to high mortality rates. The hybrid model outperformed traditional methods in accuracy, true positive rate (TPR), and precision. Results indicate this combination approach enhances heart disease prediction and aids clinical decision-making.

It is evident that combining forecasts is a crucial component in contemporary forecasting methods for temporal biomedical datasets, providing notable benefits over using single models. Nevertheless, it is crucial to thoroughly understand the data and the aim of forecasting to create an effective ensemble model. Furthermore, it is essential to employ appropriate evaluation metrics for assessing biomedical temporal forecasts. Advancements in research on efficient combination techniques may arise from the capability to manage large and varied datasets, alongside the development of automatic selection methods that balance expertise and diversity when selecting and combining models for forecasting (Wang et al., 2023).

#### 5.1.3 Coherent forecasting

This type of forecasting a.k.a. hierarchical time series (HTS) represents a set of data sequences organized by aggregation constraints, reflecting many real-world applications in research and industry. Forecasting in such hierarchical structures is challenging and time-consuming due to the need to ensure forecasting consistency among hierarchy levels based on their dimensional attributes, such as geography or product categories. Coherent forecasts are essential, meaning that higher-level forecasts must equal the sum of lower-level forecasts. This coherency requirement adds complexity to the original time series forecasting problem (Sagheer et al., 2021).

For biomedical data scenarios, HTS forecasting is applied in predicting instances similar to emergency medical services (EMS) requirements (Rostami-Tabar and Hyndman, 2024) and mortality rates across various U.S. states (Li and Hyndman, 2021; Li et al., 2024). Forecasting is crucial for EMS as it promotes consistency and synchronized resource allocation, enhancing decision-making processes and leading to better patient outcomes by avoiding the imbalance between demand and resources. In mortality rate predictions, forecasting addresses differences in mortality patterns across different geographic regions. Maintaining adherence between state-level and national-level mortality forecasts is vital for precise policy planning and resource management, aiding in reducing life expectancy disparities and enhancing public health results.

Different reconciliation procedures like top-down, bottom-up, and middle-out have been developed to maintain consistency across levels by generating base forecasts and then adjusting them. These procedures vary in approach: bottom-up starts from the lowest level and aggregates upwards, top-down begins at the highest level and disaggregates downwards, and middle-out combines both methods starting from an intermediate level. Each has its strengths and weaknesses, and none has proven universally superior. Hyndman et al. (2011) proposed an optimal combination approach, which independently forecasts all levels and then combines them using regression to ensure coherence. The Minimum Trace (MinT) method (Wickramasuriya et al., 2018) is another widely adopted approach for reconciliation. This technique uses the complete covariance matrix of forecast errors to generate a set of coherent forecasts. It aims to minimize the MSE of these coherent forecasts across the whole series, under the assumption of unbiasedness.

The approach detailed by Rostami-Tabar and Hyndman (2024) involves implementing forecast reconciliation for the hierarchical data of ambulance demand. It utilizes an ensemble of models: Exponential Smoothing State Space model (ETS), Poisson regression with Generalized Linear Model (GLM), and time series GLM (TSGLM). It generates base forecasts independently for each hierarchy level and reconcile them using the MinT method, minimizing forecast variances for coherence. Validation is done via time series cross-validation, with accuracy measured by mean absolute scaled error (MASE) and continuous ranked probability scores (CRPS). The methodology by Li and Hyndman (2021) ensures coherent mortality forecasts using a forecast reconciliation approach. Independent state-level forecasts are generated with the Lee-Carter model and then reconciled using the Minimum Trace (MinT) method together with the sampling approach by Jeon et al., (2019) to ensure consistency with national-level forecasts. Validation is performed using out-of-sample forecasting, with accuracy measured by MAPE and the Winkler score. The study uses U.S. mortality data from 1969 to 2017 and projects rates up to 2027. Another paper by Li et al. (2024) uses boosting with stochastic mortality models as weak learners. The authors extend gradient boosting with age-based and spatial shrinkage, iteratively fitting the Lee-Carter model to residuals and adding graph Laplacian-based penalties to align forecasts of adjacent age groups and states. Validation uses US male mortality data (1969–2019), with forecasting performance assessed using MASE.

Traditionally, methods like ARIMA and exponential smoothing generate base forecasts but fail to capture individual and grouped time series dynamics, especially with time variation or sudden changes. They also struggle with exploiting complete hierarchical information, affecting forecasting efficiency. Recently, ML algorithms like artificial neural networks, extreme gradient boosting, and SVR have been employed to improve accuracy by considering nonlinear relationships and dynamic changes. However, they often still rely on traditional methods and may overlook useful hierarchical information. Overall, HTS forecasting remains a complex problem with ongoing research aimed at finding more efficient and accurate methods to ensure coherent and reliable forecasts across all levels of the hierarchy (Sagheer et al., 2021). Note: A list and description of open source tools for forecasting is provided in the Supplementary Material of this article.

### 5.2 Future directions

Extensive research has been conducted to interrogate biomedical temporal data in medical and health applications. Challenges remain, and are summarized into six key areas: (1) standardizing diverse data formats; (2) managing data quality; (3) ensuring model interpretability; (4) protecting patient privacy; (5) enabling real-time monitoring; and (6) addressing bias to create fair models. To grasp the potential future developments, we present a use case to illustrate six future directions within the clinical context. Specifically, taking Mr. Smith (45 years old) as a persona who is concerned about his risk of developing Alzheimer’s Disease (AD).

#### 5.2.1 Data harmonization to standardize data format

Time series analysis plays a critical role in the early detection of AD by enabling the continuous monitoring of specific biomarkers over time. This approach is crucial for understanding the progression of the disease through its various stages, from preclinical AD to mild cognitive impairment (MCI), and ultimately to dementia. The primary biomarkers used in detecting and monitoring AD include beta-amyloid and tau proteins, which are typically measured in cerebrospinal fluid (CSF), along with imaging biomarkers such as PET scans for assessing beta-amyloid burden and MRI scans for detecting changes in brain volume. These biomarkers are indispensable for identifying the onset and progression of the disease, often before clinical symptoms become evident (Hernandez-Lorenzo et al., 2022).

During his visit to the physician, Mr. Smith is advised to undergo a series of tests, including genetic screening, neuroimaging, and cognitive assessments. These tests generate a diverse array of data types, ranging from genetic biomarkers to neuroimaging data (e.g., MRI scans) and time-series data derived from cognitive assessments. However, the data collected from Mr. Smith originate from multiple sources: a local hospital, a specialized lab for genetic testing, and a cognitive assessment app. To create a unified dataset, data harmonization is necessary, ensuring consistency across different formats, terminologies, and units. Implementing interoperable technologies can greatly facilitate seamless data exchange across disparate healthcare systems. Future research should focus on developing advanced harmonization techniques for time series data to ensure accurate and consistent integration from various sources. Additionally, integrating multi-modal data, such as clinical, genetic, and imaging information, will be crucial for creating personalized prediction models.

#### 5.2.2 Data quality

As Mr. Smith assesses his risk of developing AD, data from various tests play a critical role in forecasting his condition. However, his data may contain missing values due to irregular monitoring, different data collection protocols, or the progression of his condition. Addressing these gaps is crucial for building a reliable predictive model. A promising approach involves filling these gaps and using the missing data as a valuable signal. Missing biomarker readings can be estimated using methods like forward-filling or zero imputation. The model can also incorporate indicators to highlight absent data points, learning from the pattern of missing data. For example, if Mr. Smith's cognitive scores are missing for several months, the model can predict these values and use the absence of scores as a feature. This allows the model to detect patterns that may reveal insights such as health changes or inconsistencies in monitoring.

Ensuring data quality is essential for reliable predictive models in clinical research. Future directions should integrate advanced ML techniques that handle missing data and leverage the temporal patterns surrounding these gaps. By combining models that analyze available data and sequences of missing data, we can improve predictive accuracy, uncover hidden trends, and identify critical periods signaling disease progression. This approach enhances timely, personalized predictions for patients like Mr. Smith.

#### 5.2.3 Interpretability

As Mr. Smith assesses his risk of developing AD, advanced ML models analyzing the biomarkers to identify the intervention strategies become crucial. Current models offer predictive power but often function as “black boxes” making it challenging to understand risk factors and the associated impacts. To address this, interpretability methods are essential to know the factors behind risk predictions. One important future direction on interpretability is to use attention mechanisms that prioritize key biomarkers and time points, focusing on early disease prediction characteristics. For example, attention-based models can highlight critical data points, such as changes in biomarkers that signal the onset of AD.

A significant biomarker decline flagged by the model would make the risk assessment more transparent, aiding the physician's understanding and decisions (e.g., intervention). Alternatively, time-based SHAP (SHapley Additive exPlanations) techniques enhance model prediction transparency by assessing feature importance at specific times. Future work could focus on developing interpretability frameworks in personalized, real-time risk assessments for AD and other conditions, ensuring predictions are accurate and understandable for patients and clinicians.

#### 5.2.4 Data privacy

As Mr. Smith evaluates his risk of AD, the sensitive data gathered requires rigorous privacy safeguards. Data privacy is crucial for legal compliance and maintaining trust in the healthcare system. Sharing sensitive information in research while retaining data utility is challenging. Anonymization is a technique to safeguard against reidentification while maintaining the usefulness of research data. Blockchain technology is another method, providing secure means for sharing data. Federated learning (FL) is also beneficial for collaborative studies, enabling ML models to be trained on Mr. Smith’s data locally without the need for centralization, thus decreasing privacy risks. Informed consent is another essential aspect for research purposes. If consent is dynamic, it allows for real-time management, permitting alterations as new research develops. Future directions include implementing these techniques independently or as hybrid frameworks that improve privacy protection without sacrificing research utility. Establishing international standards for these methods is imperative for harmonizing global privacy practices and enhancing security and trust in collaborative research.

#### 5.2.5 Real-time detection

Let’s assume, the physician seeing Mr. Smith recommends the use of a wearable device that monitors essential physiological indicators such as sleep patterns and heart rate variability (HRV) to assess his AD risks. Note these devices have already demonstrated potential in identifying early signs of cognitive decline (Saif et al., 2020). With continuous, real-time monitoring, Mr. Smith would be empowered to take proactive actions—such as making lifestyle changes or seeking further medical evaluations—that could potentially delay the progression of the disease. We have observed an emerging trend in health domain to embed wearable devices into regular health surveillance, facilitating the early identification and treatment of AD or other disease conditions. A future direction in predictive modeling is high-fidelity model enabling real-time, or near real-time (e.g., 15 min) detection. Some related research questions include data storage (where data to be stored, cloud or locally), model calibration and fine tuning strategies (e.g., transfer learning).

#### 5.2.6 Bias and fairness

A typical problem in AI models is the possibility of bias if they are trained on unrepresentative datasets. For example, if a model is trained mainly on data from old Asian females, it might inaccurately evaluate Mr. Smith, who is a middle-aged American male. Future directions for utilizing AI-driven models should emphasize making these models unbiased and dependable for various populations. A critical measure is the creation and validation of AI models with datasets that include a broad spectrum of demographics, such as different ages, ethnicities, and genders. Another approach to ensure fairness in AI algorithms is through regular audits and validation by independent experts. These audits can uncover and fix biases that could distort predictions. Independent audits help guarantee that AI models are equitable and effective for diverse groups thereby offering reliable health assessments. Additionally, it is essential for both healthcare providers and patients to recognize the potential biases in AI tools. By carefully reviewing AI-generated advice alongside clinical expertise and other diagnostic tools, healthcare providers can ensure that the AI model's predictions are accurate and contextual.

## 6 Conclusion

In summary, the review paper outlines the challenges faced in predictive modeling for biomedical temporal data, such as managing missing values, addressing correlations between variables, capturing both short-term and long-term dependencies, performing multi-step ahead predictions, and considering data availability. It assesses models in three categories—statistical, machine learning, and deep learning—to evaluate their effectiveness in forecasting data amidst these challenges. Recognizing limitations in each approach, it discusses alternative methods like model enhancements or ensemble/combination forecasting techniques to potentially improve forecasting accuracy. The review also covers hierarchical forecasting for biomedical datasets with relevant structures. Moreover, it explores issues like data quality, privacy concerns, data harmonization, interpretability, real-time detection, and bias/fairness considerations in integrating AI or ML into clinical practices. These challenges underline the necessity for thorough data evaluation, strong privacy laws, and a deep understanding of the goals of predictive modeling. Moreover, successfully implementing these models necessitates a joint effort from the different fields, along with an inclusive approach that tackles not just the technical aspects of the model but also the broader ethical and fairness issues in healthcare environments.
