# Supplementary material for: Predictive modeling of biomedical temporal data in healthcare applications: review and future directions
Source: Front Physiol. 2024 Oct 15;15:1386760. doi: 10.3389/fphys.2024.1386760 (PMC11519529; doi:10.3389/fphys.2024.1386760)
Supplement: Supplementary file 1 [file DataSheet1.PDF]

Supplementary Material

1 A List and Description of Open Source Tools for Forecasting

1. **KATS:** It stands for Kits to Analyze Time Series. It is an open-source Python library by Facebook Research. It integrates with libraries like pandas, NumPy, and scikit-learn. Kats supports various forecasting models (ARIMA, Prophet, Holt-Winters, LSTM) and uses an ensemble method to enhance forecast accuracy. It offers backtesting, hyperparameter tuning, customizable pipelines, and built-in visualization tools. Kats handles large-scale time series data for real-world applications.

2. **PyFlux:** It provides a versatile interface for statistical modeling in Python. It supports models like ARIMA, GARCH, GAS, State Space, and Bayesian models, suitable for various time series data. A key feature is its Bayesian inference support, allowing prior information and uncertainty quantification. PyFlux offers tools for diagnostics, posterior distributions, forecasts, and a user-friendly API. It also supports various error term distributions, enhancing robustness.

3. **Sktime:** It is an open-source Python library for unified time series learning. It provides a unified interface for various time series tasks, supporting classical statistical methods, machine learning, and deep learning models. Its modular design allows custom pipelines by combining preprocessing, feature extraction, and modeling modules. Compatible with scikit-learn, it facilitates seamless model evaluation and hyperparameter tuning. sktime includes time series-specific transformations like detrending, differencing, and seasonal decomposition, along with robust tools for benchmarking, cross-validation, and performance metrics.

4. **Auto_TimeSeries:** It is a Python library automating time series analysis and forecasting. It handles data preprocessing, model selection, hyperparameter tuning, and evaluation. It supports ARIMA, SARIMA, Prophet, and machine learning models like Random Forest, XGBoost, and LightGBM, automatically selecting the best model and optimizing it. Ensemble methods improve forecast accuracy, and cross-validation tools ensure robust evaluation. Visualization tools aid data exploration, model diagnostics, and forecast visualization.

5. **Darts:** A Python library for manipulating and forecasting time series with models like ARIMA, Exponential Smoothing, Random Forest, XGBoost, RNNs, LSTMs, N-BEATS, and TCNs. It has an intuitive API for model building, training, and evaluation. Darts supports model ensembling, probabilistic forecasting, hierarchical forecasting, backtesting, and model evaluation. It is modular and extensible, allowing new models and features, and offers tools for data imputation and transformation.

6. **Orbit:** It is a Python package by Uber for Bayesian time series forecasting with complex seasonal patterns and holidays. It supports models like Local Linear Trend (LLT), Seasonal Local Linear Trend (SLLT), and Damped Local Trend (DLT). It is scalable and suitable for large datasets. Orbit provides probabilistic forecasts with credible intervals for better decision-making and includes tools for model evaluation and visualization.

7. **Flow Forecast:** It is a Python deep learning library for time series forecasting. It includes models like RNNs, LSTMs, GRUs, and Transformer-based models. The library offers automated hyperparameter optimization, an extensible architecture for adding models, preprocessing methods, and evaluation metrics. It supports data integration of multiple sources, enabling external features in models, and provides probabilistic forecasting for uncertainty. Users can define custom loss functions and use built-in tools for data analysis, model inspection, and forecast evaluation.

8. **Fable:** It is an R package for time series forecasting, part of the tidyverts suite. It supports models like ARIMA, ETS, and Prophet, integrating with tidyverse tools such as dplyr, tidyr, and ggplot2. Fable allows model extension and creation, supports model combination and reconciliation for hierarchical forecasts, and offers visualization tools, model diagnostics, and forecast results using ggplot2. It includes tools for backtesting and accuracy evaluation and can handle multiple time series, making it suitable for grouped or hierarchical data.

9. **ForecastComb:** It is an R package that enhances forecasting accuracy by combining multiple models. It supports methods like simple and weighted averages, regression-based approaches, principal component regression, and shrinkage estimators. ForecastComb optimizes forecast weights for the best model combination and features a user-friendly API for easy forecast merging and evaluation. It also integrates well with other R forecasting packages.

10. **ForecastHybrid:** It is an R package for combining forecasts from multiple models to enhance predictive performance. It integrates models like ARIMA, Exponential Smoothing (ETS), and Neural Networks, enabling hybrid models that leverage each technique's strengths. The package can auto-select and combine the best models based on historical data. ForecastHybrid offers probabilistic forecasting to generate prediction intervals and quantify uncertainty, aiding in decision-making. It includes tools for evaluating hybrid forecasts using accuracy metrics and cross-validation, and provides visualization tools for interpreting forecasts.
